# Changes of miRNA Expression Profiles from Cervical-Vaginal Fluid-Derived Exosomes in Response to HPV16 Infection

**DOI:** 10.1155/2020/7046894

**Published:** 2020-06-03

**Authors:** Ying Wu, Xinyan Wang, Li Meng, Wenqu Li, Chunyan Li, Ping Li, Siliang Xu

**Affiliations:** ^1^Women's Hospital of Nanjing Medical University (Nanjing Maternity and Child Health Care Hospital), Nanjing, Jiangsu, China; ^2^State Key Laboratory of Reproductive Medicine, Clinical Center of Reproductive Medicine, First Affiliated Hospital, Nanjing Medical University, Nanjing, Jiangsu, China

## Abstract

As an oncogenic virus, HPV16 can lead to the dysfunction of cervical epithelial cells and contribute to the progression of cervical cancer. Components from the cervical-vaginal fluid (CVF) could be used as the basis for cervical cancer screening. Exosomes are widely present in various body fluids and participate in intercellular communication via its cargos of proteins, mRNAs, and miRNAs. This study was conducted to explore the changes of miRNAs in exosomes isolated form the cervical-vaginal fluid during HPV16 infection and to predict the potential effects of exosomal miRNAs on the development of cervical cancer. CVF was collected from volunteers with or without HPV16 infection. The exosomes in CVF were identified by electron microscopy. Microarray analysis was subjected to find the differentially expressed miRNAs in CVF exosomes. To confirm the results, 16 miRNAs were randomly selected to go through real-time quantitative polymerase chain reaction. In addition, GO and pathway analyses were conducted to reveal potential functions of differentially expressed miRNAs. A total of 2548 conserved miRNAs were identified in the cervical-vaginal fluid-derived exosomes. In response to HPV16 infection, 45 miRNAs are significantly upregulated and 55 miRNAs are significantly downregulated (*P* < 0.05). The GO and KEGG pathway analyses revealed that these differentially expressed miRNAs are tightly associated with cervical cancer tumorigenesis, through interaction with the Notch signaling pathway, TNF signaling pathway, and TGF-*β* signaling pathway. These results suggest that exosomal miRNAs in CVF are differentially expressed in HPV16 infection patients and HPV16-free volunteers. It provided a novel insight to understand the underlying mechanism of HPV16 infection in regulating cervical cancer progression.

## 1. Introduction

Infections with certain HPV types have a high risk for cervical cancer [1, 2]. Its persistence can lead to the transformation of basal epithelial cells and contribute to the cervical cancer progression [3]. The most common carcinogenic HPV type 16 (HPV16) accounts for approximately 50% of all cervical cancers [4]. Cervical-vaginal fluid (CVF) was known to provide rich information reflecting cervical health condition. The changed components of CVF can be taken as the basis for cervical cancer screening by self-testing [5]. Notably, accumulating evidence demonstrated that abnormally high levels of mRNAs, miRNAs, and lncRNAs existed in CVF-derived exosomes [5, 6]. With the lipid bilayers, the contents of exosome in CVF can avoid RNase digestion [7]. It was recently reported that the expression of the lncRNAs HOTAIR and MALAT1 were significantly elevated in CVF-derived exosomes from HPV-positive cancer-free individuals compared to HPV-negative healthy volunteers [8]. Moreover, both of the lncRNAs have also been shown to contribute to cervical cancer progression [9, 10]. However, the changes of miRNAs in CVF-derived exosome caused by HPV16 infection and potential roles of the related miRNAs are largely unknown.

In this study, the expression profiles of miRNAs in CVF-derived exosomes from women with or without HPV16 infection were detected by the microarray technology. Some of the differentially expressed miRNAs were randomly selected and validated by quantitative reverse transcriptase PCR (qRT-PCR). Moreover, bioinformatics analysis was explored to describe the potential functions of the related miRNAs. The study on miRNAs in CVF-derived exosomes with or without HPV16 infection will help us to better understand the pathological implications of HPV16 in cervical cancer progression.

## 2. Materials and Methods

### 2.1. Collection of Cervical-Vaginal Fluid and Ethics Statement

CVF samples were collected from 6 HPV-positive and 6 HPV-negative women aged 20–35 years in Women's Hospital of Nanjing Medical University. All women had no cervical cancerous disease and abstained from sexual activity at least 3 days prior to sample collection. The samples of CVF were collected with a softcup collection device as described [11]. Then, the CVF samples were transferred into 50 mL conical centrifuge tube and were stored at −80°C until analysis. The collection procedures were approved by the Medical Ethics Committee of Women's Hospital of Nanjing Medical University. Written informed consent was obtained from all the patients.

### 2.2. Exosome Isolation

CVF samples were centrifuged at 300×*g* for 10 min followed by 2000×*g* for 30 min to remove cells and debris. The supernatants were centrifuged at 12000×*g* for 45 min to further remove cell debris and then at 100000×*g* for 70–90 min at 4°C to pellet the vesicles. Exosome pellets were resuspended in 100 *μ*L phosphate-buffered saline and stored at -80°C. All steps of centrifugation were performed at 4°C.

### 2.3. Transmission Electron Microscopy (TEM)

To observe exosome morphology, an exosome suspension was mixed with an equal volume of 2% paraformaldehyde. The mixture was subsequently applied to a formvar-coated copper grid. The sample was then stained with 1% aqueous uranyl acetate for 2 min. After being wicked off with filter paper, sample was finally observed under transmission electron microscope (FEI. Hillsboro, USA).

### 2.4. Nanoparticle Tracking Analysis (NTA)

The concentration and size distribution profile of exosomes were analyzed applying a NanoSight NS300 system (Malvern Instruments Ltd., Malvern, United Kingdom) and evaluated with NTA 3.1 Dev Build 3.1.54 software. The exosome preparations were resuspended with 800 *μ*L sterile PBS and homogenized by vortexing.

### 2.5. Western Blot Assay

Western blotting was performed to detect the presence of exosomal surface markers. We lysed CVF exosomes by RIPA lysis buffer on ice. Then, the concentration of the protein was measured using Pierce BCA Protein Assay Kit (Thermo Scientific, Massachusetts, America). Each sample was run on SDS–PAGE and transferred and blotted with exosome marker antibodies CD9 (SBI, California, America) and CD63 (SBI, California, America). The protein blots were detected by a detection system.

### 2.6. Detection and Determination of miRNAs

Total RNA was extracted and purified applying Qiagen serum/Plasma Kit (Qiagen#217184), following the instructions of the manufacturer. The RIN number to inspect RNA integration was checked applying an Agilent Bioanalyzer 2100 (Agilent Technologies, California, America). All RNA samples used for miRNA microarrays exhibited a RIN of 6.0. miRNA molecular in total RNA was labeled by miRNA Complete Labeling and Hyb Kit (Agilent Technologies, California, America) following the manufacturer's instructions and labeling section. Each slide was hybridized with 100 ng Cy3-labeled RNA using miRNA Complete Labeling and Hyb Kit (Agilent Technologies, California, America). Slides were scanned by Agilent Microarray Scanner (Agilent technologies, California, America) and Feature Extraction software 10.7 (Agilent technologies, California, America) with default settings. Raw data were normalized by Quantile algorithm, included in the R package AgimicroRNA (López-Romero, P. BMC Genomics 2011).

### 2.7. Total RNA Extraction, Reverse-Transcription Reaction, and qRT-PCR

Total RNA was obtained from exosome samples using the TRIzol reagent (Tiangen, Beijing, China) as described in the manufacturer's instructions. The quality and quantity of the extracted RNA were confirmed by the One Drop OD-1000 + Spectrophotometer (One Drop Technologies, Nanjing, China). TaqMan miRNA reverse transcriptase kit (Applied Biosystems; Thermo Fisher Scientific, Inc.) was used to synthesize cDNA with 200 ng total RNA as a template. RNA integrity was assessed by standard denaturing agarose gel electrophoresis. The qRT-PCR analysis was performed using TaqMan Universal Master Mix II no UNG (Thermo Fisher Scientific, Inc.) and commercial primers on the Applied Biosystems (Carlsbad, California, America) 7500 Real-Time PCR System. The experimental data was analyzed using the 2^−*ΔΔ*Ct^ method. All data are the average of three independent experiments.

### 2.8. GO and Pathway Analyses

Differentially expressed miRNAs were identified by a standard Student *t-*test (*P* < 0.05). The target genes of differentially expressed miRNAs were investigated in databases including TargetScan, miRDB, miRTarbase, and Tarbase. For further research, GO knowledgebase (http://www.geneontology.org) was applied to analyze biological process, cellular component, and molecular function of those predicted genes. In addition, the KEGG database (http://www.genome.jp/kegg) was applied to investigate the potential functions in the given pathways. The potential functions of differentially expressed miRNAs target genes were ranked by enrichment scores. Meanwhile, the heatmap of KEGG pathways enriched in differentially expressed miRNA was analyzed using a database (http://mpd.bioinf.uni-sb.de).

### 2.9. Statistical Analysis

Statistical differences were analyzed using Student's *t*-test or one-way analysis of variance. *P* < 0.05 was considered statistically significant. Computer-based calculations were performed with using SPSS version 20.0 statistical software.

## 3. Results

### 3.1. Characterization of Exosomes in the CVF

**Figure 1:**
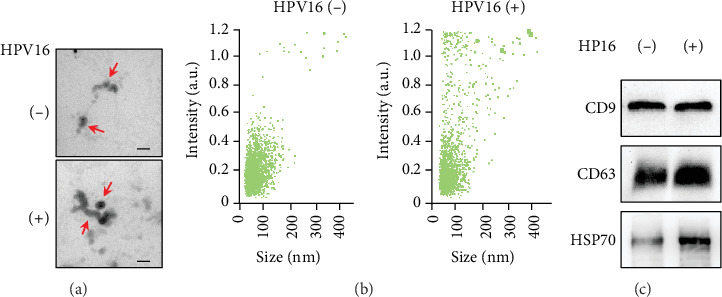
Characterization of CVF-derived exosomes. (a) Representative microscope images of CVF-derived exosome morphology were analyzed by electron microscopy. Scale bar, 200 nm. (b) Exosomal average size and intensity were measured through NanoSight analysis. (c). Expression of exosome markers CD9, CD63, and HSP70 was detected via western blot.

TEM, NTA, and western blot analysis were used to examined and confirm the exosomes isolated from CVF. Under TEM, round vesicle structures with sizes varying between 30 and 150 nm were observed (Figure 1(a)). NTA showed that the size distribution peaking was located at about 100 nm diameter (Figure 1(b)), consistent with the previously reported characteristics of exosomes [12]. Exosomal membranes are rich of endosome-specific markers, such as CD9, CD63, and CD81 [13]. The results of western blot showed that CD9 and CD63 were both present in CVF-derived exosomes (Figure 1(c)). All these data indicated the successful isolation of exosomes from CVF.

### 3.2. Differential Expression of Exosomal miRNAs in Response to HPV16 Infection

**Figure 2:**
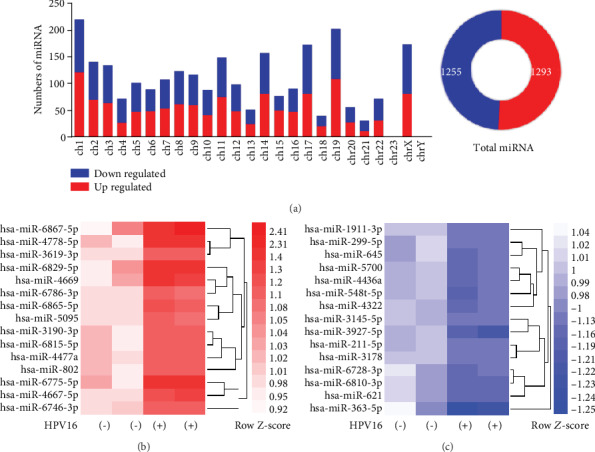
Differentially expressed miRNAs in exosomes of HPV16-infected CVF and control samples. (a) Upregulated or downregulated miRNAs were marked in red or blue, respectively. The number of all conserved miRNAs located in the human chromosomes was shown in the histogram. The circular diagram on the right represents total conserved miRNAs in the human chromosome, with 1293 upregulated and 1255 downregulated. Heatmap analysis showing the fold change of the top 15 (b) highest and C. lowest miRNAs in the HPV16-infected group. Color scale is from -1.25 (blue, lower than mean) to 2.41 (red, higher than the mean). Each column represents one sample, and each row indicates a transcript.

The microarray technology was used to compare miRNA expression profiles of CVF-derived exosomes between HPV16-positive and HPV16-negative women. Based on the results of the microarray analysis, 2548 miRNAs from various chromosomes were found. Mostly, deregulated miRNAs were located in chromosome 1, 19, and X (Figure 2(a)). In total, 55 downregulated and 45 upregulated miRNAs satisfied the significant threshold in the HPV16-positive group (*P* < 0.05). These significantly differentially expressed miRNAs can be found in Supplementary Table 1. Top 15 significantly upregulated miRNAs included hsa-miR-6865-5p, hsa-miR-3190-3p, hsa-miR-6815-5p, hsa-miR-802, and so on (Figure 2(b)). Top 15 significantly downregulated miRNAs included hsa-miR-363-5p, hsa-miR-548 t-5p, hsa-miR-621, hsa-miR-645, and so on (Figure 2(c)).

### 3.3. Validation of Differentially Expressed miRNAs by qRT-PCR

**Figure 3:**
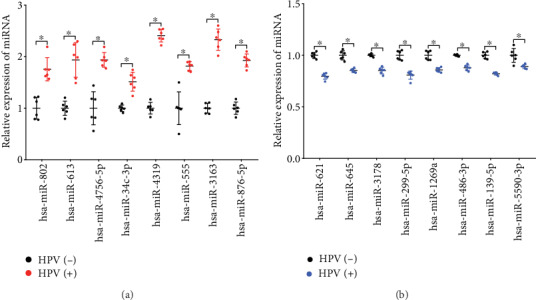
Validation of the differentially expressed miRNA using qRT-PCR. (a). Eight significantly upregulated miRNAs were randomly selected and validated in exosomes of HPV16-infected CVF and control samples by qRT-PCR. (b). Eight significantly downregulated miRNAs were also validated.

To confirm the differential expression of miRNAs, 16 different miRNAs were randomly selected and qRT-PCR was conducted. The expression patterns of these dysregulated miRNAs in HPV-16 infected CVF exosomes shows consistency with the results of microarray analysis (Figures 3(a) and 3(b)). The qPCR primers used in this study can be found in Supplementary Table 2.

### 3.4. GO Enrichment and KEGG Pathway Analyses of Differentially Expressed miRNAs Target Genes

**Figure 4:**
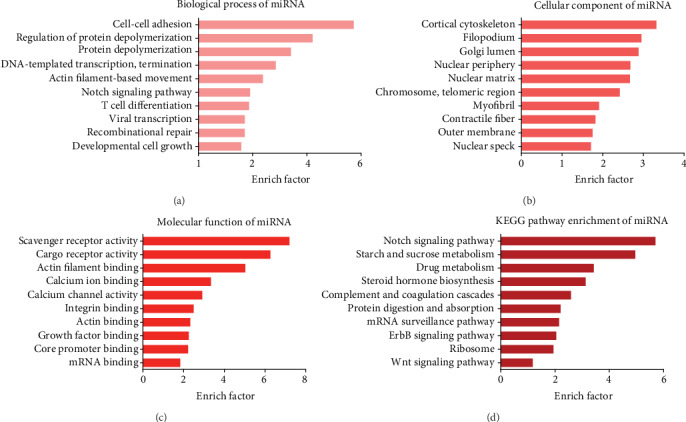
Potential function of differentially expressed miRNA. GO and KEGG analyses of differential genes can identify functional or metabolic pathways of differentially expressed miRNAs. Elucidate differences between samples at the level of gene function and metabolic pathways. According to the function annotation of genes, the number of differential genes belonging to different functions is displayed in the form of a bar graph. GO knowledgebase was applied to analyze biological process, cellular component. and molecular function of miRNA predicted genes. KEGG database was applied to investigate the potential functions in the given pathways. (a). The biological process categories. (b). The cellular component categories. (c). The molecular function categories. (d). KEGG signaling pathways. Line length indicates the strength of data support.

**Figure 5:**
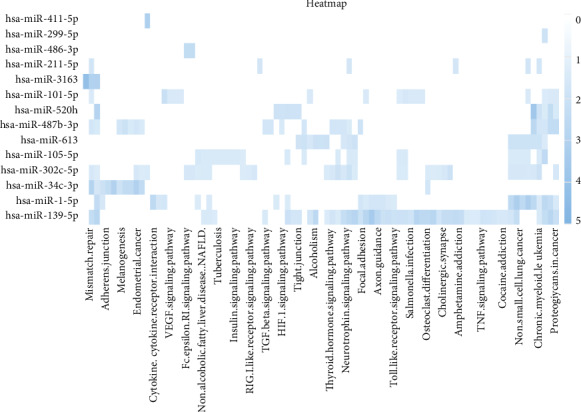
Heatmap of KEGG pathways. KEGG pathways of differentially expressed miRNA were involved in multiple pathways, especially proteoglycans in cancer, TNF signaling pathway, and TGF-*β* signaling pathway. The intensity increases from white to blue.

GO and KEGG pathway analyses were applied to reveal potential functions of differentially expressed miRNA target genes (listed in Supplementary Table 3). The targets genes are involved in various biological processes, including cell proliferation, recombination repair, viral transcription, and T cell differentiation. (Figure 4(a)). Nuclear speck, outer membrane, contractile fiber, myofibril, etc., were the highly enriched cellular component (Figure 4(b)). mRNA binding, growth factor binding, actin binding, integrin binding, etc., were the highly enriched molecular function (Figure 4(c)). The KEGG pathway analysis revealed that the differentially expressed miRNA target genes were involved in Wnt signaling pathway, ErbB signaling pathway, and Notch signaling pathway (Figure 4(d)). In addition, the KEGG pathway heatmap of differentially expressed miRNAs were also conducted. The KEGG pathway, cross talking with multiple pathways, especially proteoglycans in cancer, TNF signaling pathway and TGF-*β* signaling pathway, may contribute to tumorigenesis/cancer progression (Figure 5).

## 4. Discussion

The persistent infection of HPV is causally linked with the development of cervical cancer [14]. Among the twelve high-risk HPV genotypes (HPV16, 18, 31, 33, 35, 39, 45, 51, 52, 56, 58, and 59), HPV16 is the most carcinogenic HPV type [15]. HPV16 is a small nonenveloped virus with double-stranded DNA as its genetic material [6]. It was reported that E6 and E7, as important functional coding regions of HPV16, were the most responsible oncoproteins for their tumorigenesis [16]. The E6 protein promoted abnormal cell growth by rapidly targeting p53 protein through direct binding [17]. The E7 protein encoded by high-risk HPVs can immortalize human epithelial cells and contribute to the development of cervical carcinoma. [18]. After HPV infection, the miRNA profiles of CVF-derived exosomes change to obtain the carcinogenic feature. A recent study revealed that miRNA-21 was observed to be upregulated in HPV18-infected CVF-derived exosomes [19]. Notably, miRNA-21 was reported to promote cell growth, migration, and invasion of human cervical cancer cells. In addition, several miRNAs also showed oncogenic potential and played a major role in cervical cancer development. For example, it was reported that miR-3156-3p played a tumor-suppressor role in cervical cancer and its downregulation was associated with cervical cancer pathogenesis through the promotion of cell proliferation, migration, invasion, and tube formation [20]. miR-10b was found to be markedly downregulated during cervical cancer progression, and the lower miR-10b level in cervical cancer was positively associated with the more aggressive tumor phenotype [21]. However, there has been limited research on the screening and sequencing of the exosomal miRNAs in response to HPV16 infection. In our study, we speculate that miRNAs in CVF-derived exosomes may participate in the development of cervical cancer after HPV infection. Whether miRNAs in CVF-derived exosomes during HPV16 infection can be used as a cofactor of cervical cancers needs to be further determined.

According to the results of microarray assays, there were 45 upregulated miRNAs and 55 downregulated miRNAs in HPV16-infected CVF-derived exosomes compared to the uninfected samples. Among these, hsa-miR-5590-3p was the most significantly downregulated miRNA. Previous studies have demonstrated that hsa-miR-5590-3p acted as a negative regulator of the TGF*β*/SMAD signaling pathway through the downregulation of TGF*β*-R1 and SMAD4 transcripts [22]. However, the expression of TGF-*β*1 and SMAD4 proteins was elevated in cervical adenocarcinoma tissues and cervical squamous carcinoma [23]. Despite the downregulated miRNAs, the significantly upregulated miRNAs after HPV16 infection may also participate in the progression of cancer. For example, miR-613 was upregulated in colon cancer tissue samples, which also promoted the proliferation, invasion, and migration of colon cancer cells [24]. miR-27b-5p was reported to be overexpressed in gastric low-/high-grade dysplasia and might play an important role in gastric cancer development from premalignant adenomas [25]. Our data suggests that these changed miRNAs showed oncogenic potential and may provide help to understand the pathogenesis of HPV16 infection in cervical cancer.

Notably, bioinformatics analysis of differentially expressed miRNAs target genes revealed that these miRNAs were also actively involved in oncogenic pathways through targeting specific genes. The results of GO analysis demonstrated that the differentially expressed miRNAs target genes such as RPL14, NUP205, and POLR2B were associated with viral transcription, which is a typical feature of HPV infection. Moreover, the results of the KEGG pathways including the Notch signaling pathway, ErbB signaling pathway, and Wnt signaling pathway were mainly associated with the progression of cervical cancer. the Notch signaling pathway was demonstrated to be highly correlated with carcinogenesis, including the cervical cancer development [26]. ErbB2, as a key protein in ErbB signaling pathway, was demonstrated to act as a poor prognostic factor in human cervical cancer and considered as a target for cervical cancer therapy [27]. Furthermore, it has been shown that cervical tumorigenesis is a multistep process, and the activation of Wnt/*β*-catenin pathway was a first hit [28]. E6/E7 oncoproteins of HPV16 are reported to regulate nuclear accumulation of *β*-catenin and thereby regulates the Wnt signaling pathway [29]. In addition, the KEGG heatmap of the differentially expressed miRNAs were involved in multiple pathways, including proteoglycans in cancer, TNF signaling pathway, and TGF-*β* signaling pathway. The TNF signaling pathway is known to be actively associated with inflammation. HPV-infected cells could exhibit activation of NF-*κ*B and STAT3, which are mediators of inflammation [30]. Emerging evidence suggests that inflammation plays a key role in the initiation and progression of various cancers, including cervical cancer [31]. These results suggest that these altered miRNAs may play important roles in the oncogenic process of HPV16 infection.

In conclusion, the expression of miRNAs in CVF-derived exosomes was significantly different after HPV16 infection. Bioinformatics analysis of the dysregulated miRNAs showed that these differentially expressed miRNAs may be tightly involved in the progression of oncogenesis. Our study provides a foundation for understanding the mechanism of the oncogenic process of HPV16 infection. We will expand the sample size and explore the detailed mechanism of these miRNA changes. Further studies will be proceeded to evaluate whether the exosomal miRNA in CVF can potentially serve as useful biomarkers for cervical cancer diagnosis.

## Data Availability

The data that support the findings of this study are available from the corresponding author upon reasonable request.

## Supplementary Materials

Supplementary materials Supplementary Table 1: all of the differentially expressed miRNAs. Supplementary Table 2: list of qPCR primers used in this study. Supplementary Table 3: target genes of differentially expressed miRNAs.
